# The establishment of a recombinase polymerase amplification technique for the detection of mouse poxvirus

**DOI:** 10.1186/s12917-023-03703-3

**Published:** 2023-12-06

**Authors:** Yuexiao Lian, Mengdi Zhang, Yujun Zhu, Miaoli Wu, Bihong Huang, Li Xiao, Kehang Shi, Peide Li, Feng Cong, Huanan Wang

**Affiliations:** 1 Guangdong laboratory animals monitoring instituteand Guangdong Provincial Key Laboratory of Laboratory Animals, Guangzhou, 510663 China; 2 Department of Veterinary Medicine, College of Animal Sciences, Zhejiang University, Hangzhou, 310058 China; 3 MOA Key Laboratory of Animal Virology, Center for Veterinary Sciences, Zhejiang University, Hangzhou, 310058 China; 4 Wenzhou Engineering Research Center of Pet, Department of Animal Science, Wenzhou Vocational College of Science & Technology, Wenzhou, 325006 China

**Keywords:** Ectromelia virus, Recombinase polymerase amplification, Detection, *CrmD* gene, Qualitative analysis

## Abstract

**Background:**

Ectromelia virus (ECTV) is the causative agent of mousepox in mice. In the past century, ECTV was a serious threat to laboratory mouse colonies worldwide. Recombinase polymerase amplification (RPA), which is widely used in virus detection, is an isothermal amplification method.

**Results:**

In this study, a probe-based RPA detection method was established for rapid and sensitive detection of ECTV.Primers were designed for the highly conserved region of the *crmD* gene, the main core protein of recessive poxvirus, and standard plasmids were constructed. The lowest detection limit of the ECTV RT- RPA assay was 100 copies of DNA mol-ecules per reaction. In addition, the method showed high specificity and did not cross-react with other common mouse viruses.Therefore, the practicability of the RPA method in the field was confirmed by the detection of 135 clinical samples. The real-time RPA assay was very similar to the ECTV real-time PCR assay, with 100% agreement.

**Conclusions:**

In conclusion, this RPA assay offers a novel alternative for the simple, sensitive, and specific identification of ECTV, especially in low-resource settings.

## Background

Ectromelia virus (ECTV) is an orthopoxvirus whose natural host is the mouse [1]. It was first discovered in 1930 when mice with the disease had obvious characteristics of foot loss found in the experiment [2]. Subsequently, The disease occurred and was prevalent in mouse colonies in Europe, Japan, China, and the United States [3–5]. ECTV can be transmitted by infecting mouse foot pads. The typical clinical manifestations of mice infected with ECTV were swelling, ulceration and necrosis of limbs, head and tail, and later symptoms of toe falling off [6]. ECT is characterized by the fulminant epidemic, high fatality rate and serious harm to the production and scientific research of laboratory animals. This led to a multi-million dollar loss in biomedical research funds [7].Consequently, mousepox is one of the pathogens that must be excluded in Specific Pathogen Free (SPF) mice.Accordingly it is crucial to confirm the ECTV-free status of mice in the laboratory. Notably, mousepox outbreaks in laboratory mouse colonies have been eliminated owing to environmental strict surveillance and SPF mice improvements in the animals [7] but infections continue to occur sporadically [8]. Therefore, it is necessary to establish a rapid and sensitive virus detection and diagnosis system to prevent and control ECTV infection.

Most traditional ECTV detection methods are clinical diagnoses that use electron microscopic observation of pathological tissue sections, virus isolation, and serological tests [9–12]. However, these methods are time-consuming and require stringent experimental conditions. Hence, they are unsuitable for large-scale detection. Furthermore, molecular biological methods, such as conventional PCR and fluorescence quantitative PCR, are the most commonly used qualitative and quantitative detection methods for pathogens [13]. These methods require sophisticated instrumentation and experienced technicians. Therefore, it is very important to develop a rapid, sensitive and effective method.

Recombinase polymerase amplification (RPA) is a new isothermal amplification technique, which was first used in2006 [14]. This method simulates the amplification process of phage gene replication under the action of three enzymes, and uses recombinase instead of the thermal cycle required by PCR. It uses single-stranded binding proteins to anneal primers and strain-displacement DNA polymerases to amplify nucleic acid sequences [14]. The technique requires only two primers and takes no more than 30 min to detect [15]. In addition, RPA technology is simple to operate, has low requirements on equipment, and has high specificity and sensitivity; it is also used to detect a variety of pathogens [16–19]. To the best of our knowledge, there have been no reports using RPA as a diagnostic tool for ECTV in mice.

In this study, a sensitive and specific RPA detection method was established, which can effectively and rapidly diagnose ECTV. This is of great significance for the prevention and control of disease transmission.

## Methods

### Viruses

Murine norovirus, mouse hepatitis virus A59 strain (ATCC Product No.VR-764), sendai virus Sendai/52 strain (ATCC Product No.VR-105), Ectromelia virus (ATCC Product No.VR-1374), pneumonia virus of mice (ATCC Product No.VR-25), and reovirus type3 (ATCC Product No.VR-824, BHK-21 (ATCC CCL-10) were preserved in the Guangdong Laboratory Animal Monitoring Institute.

### Generation of the DNA molecule standard

To generate a molecular standard that can be used as a positive control for experiments and a calibration template with known copy numbers, the genomic DNA of ECTV was extracted from the virus using the TIA Namp Virus genomic DNA/RNA kit (TIANGEN Biotech, Beijing, China) according to manufacturer’s requirement. The sense primer was ECTV- (5′ TGACTCATTCCTCTAATACCACTTCTAATA-3’), and the reverse primer was ECTV-R (5′ACTACACGATTTAGTCTCTACTTGATTACT3’). The *CrmD* gene (GenBank accession number: OU343159) fragment was amplified using purified DNA as the template, and it was cloned into the pMd-19T vector (Takara, Dalian, China) and transformed into *E. coli* DH5α (Takara, China). The plasmid DNA concentrations were measured using the Nano2000 (GE, USA) and the subsequently converted copy number was calculated. The dilution range of the standard was 10^0^–10^6^ copies/ µL in order to help assess the sensitivity of the RPA assay.

### Primers and probe design

At present, there is no RPA primer and probe design software. First, RPA primers were designed to amplify the conserved gene.Then, according to the instructions in the TwistDx manual, an RPA Exo probe wwas designed between the forward and reverse primers to demonstrate the specificity of the primers.Both primers and probes were provided by Guangzhou Biological CO., Ltd. (Guangzhou, China). All of the primer and probe sequences were screened for performance using RPA, as shown in Table 1.

**Table 1:** Primers and probe sequences for the RPA detection of ECTV

Name	Sequence (5’-3’)	Amplicon size
ECTV-1 F	AAAAGTGTCCGGGTAATATAGATAAATGTG	144 bp
ECTV-1R	GTAATTGTTAACTCGGAAGTTGATATGGTA	
ECTV-2 F	TACCATATCAACTTCCGAGTTAACAATTAC	129 bp
ECTV-2R	ATGTACTTTATCGTTTGTAAAGAAACCTGA	
ECTV-3 F	TGACTCATTCCTGTAATACCACTTCTAATA	145 bp
ECTV-3R	ACTACACGATTTAGTCTCTACTTGATTACT	
Probe	GATGACACCTTTACATCCATTCCTAATCA C/FAM/THF/g/BHQ1/CCCGCGTGTCTAAG	

### Real-time RPA assay

Using the Twist Amp TM lyophilized kit (Twist DX, Cambridge, United Kingdom), The reagents were used for target primer screening. The RPA reaction volume was 50 µL containing a tube of reactive dry powder, 25 µL of Buffer A, 2 µL of ECTV-F/R primers (10 µmol/L), 13.2 µL of water, and 5 µL of the template. The premixed solution was transferred to a reaction tube containing lyophilized powder for RPA amplification. Then 2.5 µL of magnesium acetate was added to the tube’s cap.The reaction tubes were completely inverted and briefly centrifuged. The microtubes were immediately placed in a heating block and incubated at 37 °C for 30 min. The final RPA products were electrophoresed on 2% agarose gels.

The real-time RPA reaction Twist Amp TM exo lyophilized kit (Twist DX, Cambridge, United Kingdom) was used according to the manufacturer′s instruction. The reaction assay of the real-time RPA was similar to the basic RPA, and 0.6 µL of the probe replaced 0.6 µL of molecular grade H_2_O.After mixing and instant centrifugation, the reaction was started by placing the tubes in a constant temperature fluorescence detector (DEAOU Biotechnology, China) at 37 ° C for 30 min. All real-time RPA responses were repeated three times.All of the samples that had exponential amplification curves with a cycle threshold above the threshold of the negative control were considered positive.

### Sensitivity and specificity of the assay

To determine the sensitivity of the assay, a 10-fold serial dilution of the ECTV DNA standard ranging from 10^0^ copies/µL-10^6^ copies/µL was tested using the assay in three replicates to determine the sensitivity of the RPA assay. The specificity of the real-time RPA assay towards ECTV was assessed using several highly prevalent infectious pathogens in mice. Each run included ECTV positive samples and ultrapure water negative controls.

### Clinical samples

To verify the applicability of this method, real-time RPA was performed on DNA extracts from 135 mouse (BALB/C) tissue samples. The mice were from Guangdong Medical Lab Animal Center.The clinical samples of ECTV are relatively small, some are collected from various places, and some are tissue samples from animal infection experiments. The mice were euthanized with the cervical dislocation. All the animal procedures were approved by the Office of Laboratory Animals. The total nucleic acids of the specimens were extracted using a TIA Namp Virus genomic DNA/RNA kit (TIANGEN Biotech, Beijing, China) and tested using bothreal-time RPA and real-time PCR [20]. For comparison, the template and total volume of the two tests were the same.

## Results

### Selection of the primer-probe set

**Fig. 1:**
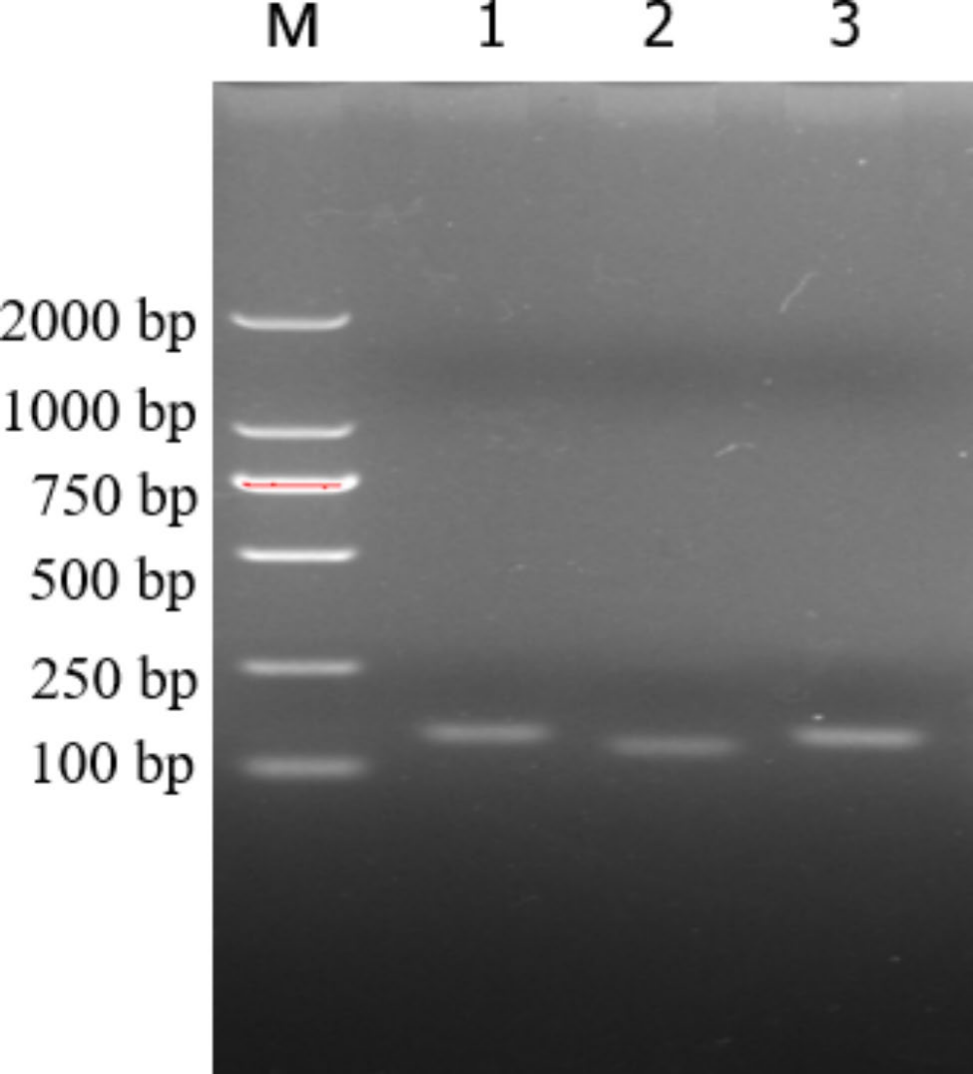
The products of the real time RPA using three pairs of primers were subjected to agarose gel electrophoresis. The F3/R3 set and the probe were the best combination and were used for the further assessment of the RPA assay. Number 1 represent primer 1,Number 2 represent primer 2,Number 3represent primer 3

Since the optimal primer/probe combination for RPA could not be predicted a priori, a preliminary screening of primers and probes was performed to select a set of effective primer probes. A total of three forward primers and three reverse primers were initially designed. The primer set F1/R1 F2/R2 F3/R3 successfully amplified the target region (Fig. 1).The primer set F3/F3 emitted the strongest light under UV irradiation.Therefore, primer combination F3/R3 was used to establish a real-time RPA detection method for ECT detection(Fig. 4). The sequences of primer candidates and probes used in this study are included in Table 1.

**Fig. 2:**
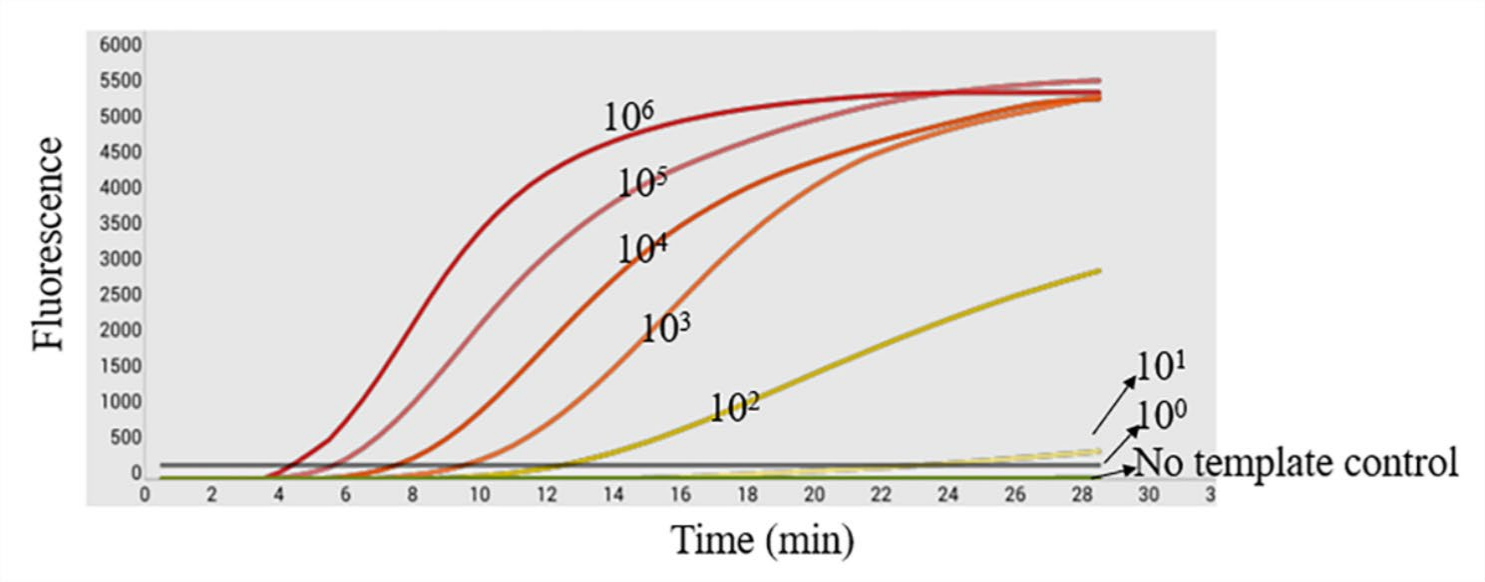
Sensitivity analysis of the RPA assay. The sensitivity of the Real-timeRPA assay was assessed by testing the serial diluted DNA standards ranging from 1 × 10^6^ copies/µL to 1 × 10^0^ copies/µL, the detection limit was 1 × 10^2^copies/µL

**Fig. 3:**
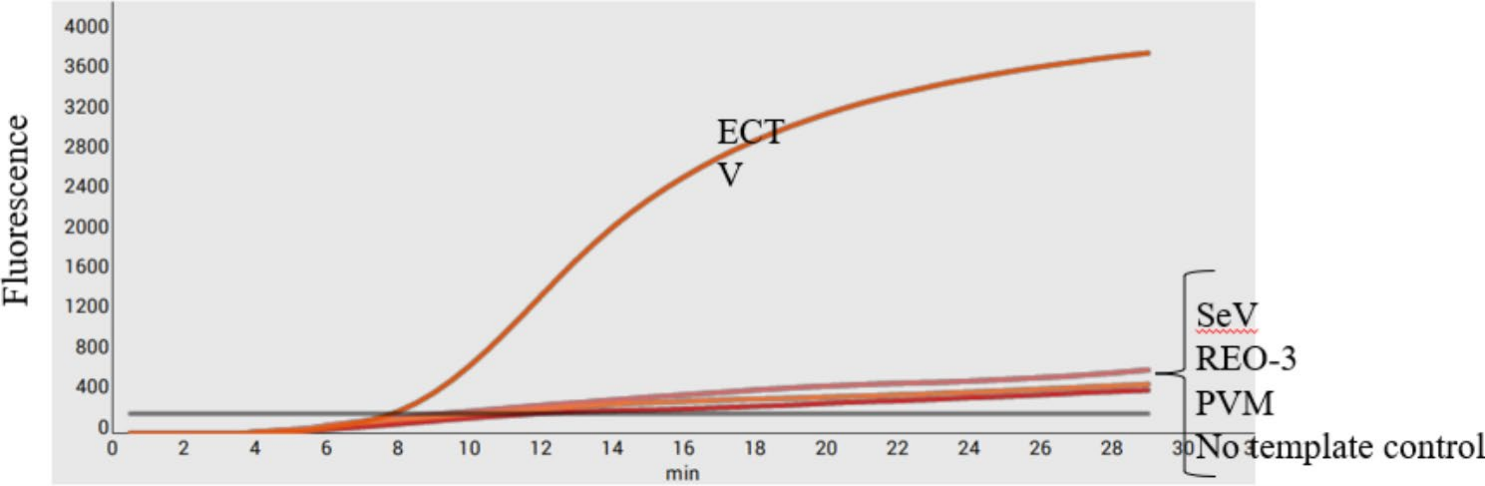
Specificity of the RT-RPA assay. The specific fluorescent signals were detected from Ectromelia virus (ECTV) DNA using the RPA method, and no fluorescence signals with Murine norovirus, mouse hepatitis virus A59 strain, sendai virus Sendai/52 strain, pneumonia virus of mice, and reovirus type3 were observed. Nuclease free water was used as a negative control. The results showed that the RPA assay was highly specific

### Analytical sensitivity and specificity

To evaluate the sensitivity of the real-time RPA assay for ECTV, a ten-fold serial dilution of the ECTV DNA standard ranging from 10^0^ copies/µL–10^6^ copies/µL was detected. The result showed that the detection limit of ECTV using the RPA assay was 100 genomic copies per reaction. The assay detected up to 10^2^ copies/µL of the target DNA **(**Fig. 2). A semi-logarithmic regression analysis was performed using the data from the sensitivity results of the standard samples, and the correlation coefficient (R^2^) was 0.9802 (Fig. 5). In this study, genomes from other common murine viral pathogens were examined using the real-time assay. ECTV DNA stock and ultrapure water wererespectively used as positive and negative controls. The amplification specific curve was only observed for ECTV, and the other viruses and the negative control displayed no specific curves (Fig. 3). This indicates that the method has high specificity for ECTV.

### Performance of the RPA assay on clinical samples

The actual performance of the RPA detection system was further verified in clinical samples. We collected 135 murine tissue samples, All samples were detected by real-time RPA and real-time PCR. Of these samples, 26 ECTV-positive and 109 ECTV-negative samples were confirmed by real-time RPA. The real time PCR result showed that 26 samples were ECTV positive, and 135 samples were ECTV negative. The detailed test results are displayed in Table 2.

**Table 2:** Comparison of the results of the real-time RPA and the real-time PCR

		Real-time PCR			
		Positive	Negative	Total	CR
Real-time RPA	Positive	26	0	26	100%
	Negative	0	109	109	
	Total	26	109	135	

## Discussion

ECTV is the causative agent of murinepox, an acute systemic disease with high mortality in susceptible strains of mice, and this virus is used as a model to study orthopoxvirus infection [21, 22]. Mousepox has been a serious threat to laboratory mouse colonies in the past [7, 23, 24]. Developments and improvements in animal husbandry and disease surveillance have reduced the incidence of ECTV. However,routine health monitoring of rodents is vital. Therefore, a rapid and sensitive method for detecting ECTV infection is important for quality control and health monitoring of laboratory mice.

Virus isolation in cell culture is considered to be the gold standard method for detection and confirmation of infection [25]. Viral protein immunoblotting is a method of detecting viruses. At present, molecular methods, such as real-time polymerase chain reaction, are widely used for the detection and quantification of poxviruses due to several advantages. Previously validated assays have demonstrated that many poxvirus genes can be used for real-time PCR assays [26–28]. However, this approach is complex and expensive. Compared with those methods, the real-time RPA assay developed in this study offers several advantages. These advantages include the following:RPA reagents can be lyophilized with the primary RPA reagents provided in a single dried pellet; assay preparation is simplified; and contamination is reduced. These allow for easy transportation and long-term storage of the reagents at room temperature [29]. Another advantage of isothermal assays is that RPA assays can withstand a wide range of reaction temperatures and do not require precise temperature control [30]. Previous studies have shown that RPA retains reliable functionality between 31 and 43 °C [31] and some have shown even between 30 and 45 °C [32, 33]. The technique can accomplish exponentially amplified target sequences in 30 min at relatively low temperatures, making it suitable for use in resource-poor situations with minimal training and equipment.

This study successfully established a simple and rapid real-time RPA detection method for ECTV detection [15]. Primer-probe set was designed based on highly conserved *CrmD* gene sequences. Three primer pairs were screened for the optimal performance of the assay. The primer pair 3 F/3R produced a strong gene amplification signal. Furthermore, the probe was designed based on the target region of the primer set 3 F/3R. The primer-probe set produced a satisfactory result. Thus, the optimal primer pair 3 F/3R was used in the RPA assay. Only the nucleic acid from ECTV could be amplified by the assay, suggesting the system had no cross-reaction with other common murine viruses and possessed remarkable specificity. The sensitivity of this was 100 genomic copies/µL. The correlation between the two methods shows that there was a significant linear relationship between the logarithm of the plasmid concentration and the Tt value for ECTV with the R^2^ values of 0.9802. This indicated the potential quantitative detection capability of this established diagnosis platform.

135 field samples were used to evaluate the clinical performance of real-time RPA detection. The results of the real time RPA and real time PCR testing of the sample were similar. The comparative experimental results show that the real-time RPA developed in this study can reliably detect ECTV. Using a simple low-cost RPA technique for molecular diagnosis of diseases is an alternative approach in resource-limited situations.This can lead to immediate treatment of positive cases in the field, following to timely diagnosis and confirmation.

**Fig. 4:**
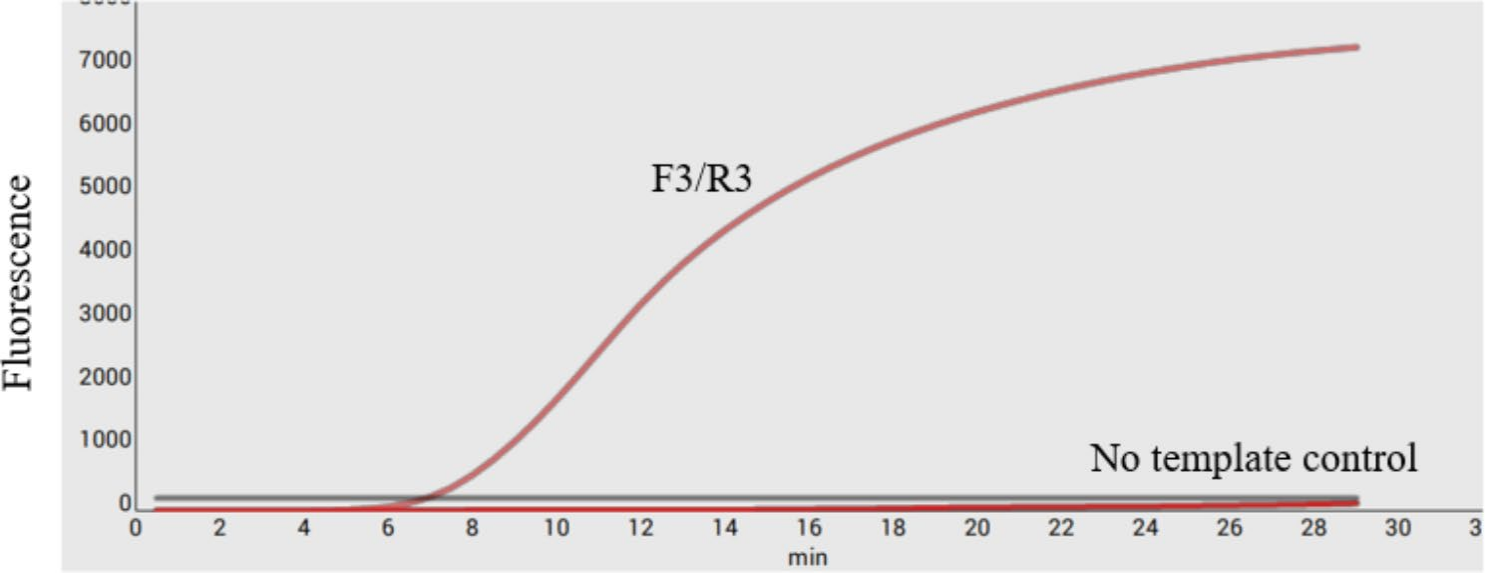
The primer pairs were evaluated using the probe-based real time RPA assay

**Fig. 5:**
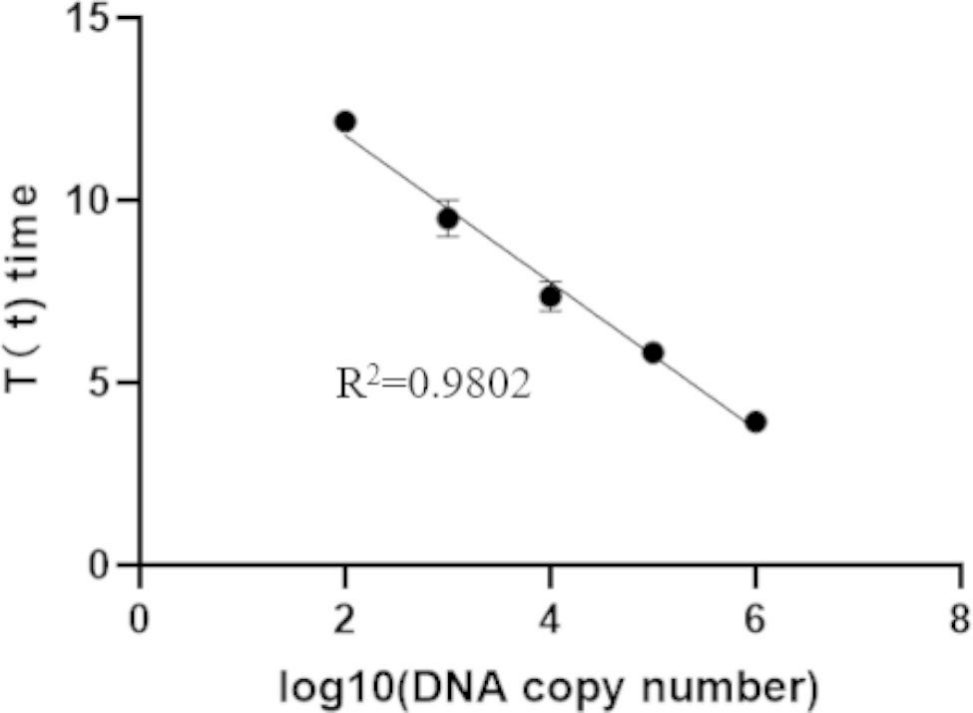
Semi-logarithmic regression of the Tt values and standards concentration using GraphPad Prism 8.0

## Conclusion

A real-time RPA assay was developed to detect ECTV DNA in samples quickly and accurately, with high sensitivity and specificity.The method works faster than existing molecular methods for detecting ECTV, with a running time of 30 min. This assay provided an alternative diagnostic method for laboratory animals and has the potential for practical applications in field detections.

## Data Availability

All data generated or analyzed during the study are included in this published article.

## Abbreviations

ECTV: Ectromelia virus
RPA: Recombinase polymerase amplification
SPF: Specific Pathogen Free
